# IQGAP1 binds AMPK and is required for maximum AMPK activation

**DOI:** 10.1074/jbc.RA120.016193

**Published:** 2020-11-21

**Authors:** Andrew C. Hedman, Zhigang Li, Laëtitia Gorisse, Swetha Parvathaneni, Chase J. Morgan, David B. Sacks

**Affiliations:** Department of Laboratory Medicine, National Institutes of Health, Bethesda, Maryland, USA

**Keywords:** AMP-activated kinase (AMPK), scaffold protein, signaling, protein–protein interaction, metformin, metabolic regulation, homeostasis, calcium, calmodulin (CaM), IQGAP1, ACC1, acetyl-CoA carboxylase, AMPK, AMP-activated protein kinase, CaM, calmodulin, CaMKK2, Ca^2+^/calmodulin-dependent protein kinase kinase 2, CaMK1, Ca^2+^/calmodulin-dependent protein kinase 1, [Ca^2+^]_i_, intracellular free Ca^2+^ concentration, DMEM, Dulbecco’s modified Eagle’s medium, ERK, extracellular signal-regulated kinase, FASN, fatty acid synthase, FBS, fetal bovine serum, G6PC, glucose-6-phosphatase, IQGAP1, IQ motif–containing GTPase-activating protein, LKB1, liver kinase B1, MAPK, mitogen-activated protein kinase, MEF, mouse embryonic fibroblast, NIRS, nonimmune rabbit serum, PCK1, phosphoenolpyruvate carboxykinase, PI3K, phosphoinositide-3-kinase, PVDF, polyvinylidene difluoride

## Abstract

AMP-activated protein kinase (AMPK) is a fundamental component of a protein kinase cascade that is an energy sensor. AMPK maintains energy homeostasis in the cell by promoting catabolic and inhibiting anabolic pathways. Activation of AMPK requires phosphorylation by the liver kinase B1 or by the Ca^2+^/calmodulin-dependent protein kinase 2 (CaMKK2). The scaffold protein IQGAP1 regulates intracellular signaling pathways, such as the mitogen-activated protein kinase and AKT signaling cascades. Recent work implicates the participation of IQGAP1 in metabolic function, but the molecular mechanisms underlying these effects are poorly understood. Here, using several approaches including binding analysis with fusion proteins, siRNA-mediated gene silencing, RT-PCR, and knockout mice, we investigated whether IQGAP1 modulates AMPK signaling. *In vitro* analysis reveals that IQGAP1 binds directly to the α1 subunit of AMPK. In addition, we observed a direct interaction between IQGAP1 and CaMKK2, which is mediated by the IQ domain of IQGAP1. Both CaMKK2 and AMPK associate with IQGAP1 in cells. The ability of metformin and increased intracellular free Ca^2+^ concentrations to activate AMPK is reduced in cells lacking IQGAP1. Importantly, Ca^2+^-stimulated AMPK phosphorylation was rescued by re-expression of IQGAP1 in IQGAP1-null cell lines. Comparison of the fasting response in wild-type and IQGAP1-null mice revealed that transcriptional regulation of the gluconeogenesis genes PCK1 and G6PC and the fatty acid synthesis genes FASN and ACC1 is impaired in IQGAP1-null mice. Our data disclose a previously unidentified functional interaction between IQGAP1 and AMPK and suggest that IQGAP1 modulates AMPK signaling.

The AMP-activated protein kinase (AMPK) regulates signaling pathways to maintain cellular energy demands. AMPK is a heterotrimer comprising three subunits: an α kinase subunit (which exists as α1 or α2 isoforms in mammals) and the β and γ regulatory subunits. AMPK is usually active only when phosphorylated at Thr-172 on the α subunit (1). Phosphorylation of Thr-172 is mediated by one of two kinases: namely liver kinase B1 (LKB1) (2) and Ca^2+^/calmodulin-dependent protein kinase 2 (CaMKK2) (3, 4, 5). Energy stress that reduces ATP production (*e.g.*, hypoxia or inhibition of glycolysis) or promotes ATP consumption (*e.g.*, skeletal muscle contraction) increases the ratio of ADP:ATP and AMP:ATP. The increased ADP and AMP enhance Thr-172 phosphorylation of AMPK by LKB1. Generally, LKB1 mediates AMPK phosphorylation in response to nutrient deprivation or metformin treatment in most cell types (6). By contrast, CaMKK2 is activated by Ca^2+^. When intracellular free Ca^2+^ concentrations ([Ca^2+^]_*i*_) increase, Ca^2+^ binds to and activates the Ca^2+^ regulatory protein calmodulin (CaM). Ca^2+^/CaM then binds to and activates CaMKK2, which catalyzes phosphorylation of AMPK (3). Once activated, AMPK catalyzes the phosphorylation of its substrates, such as acetyl-CoA carboxylase 1 (ACC1), 3-hydroxy-3-methylglutaryl-Co enzyme A reductase (HMGCR), and sterol regulatory element-binding protein 1 (SREBP1) (7, 8), to modulate processes that promote catabolic and inhibit anabolic pathways (reviewed in (9, 10, 11)). These processes include promoting the surface localization of GLUT1 and GLUT4 glucose transporters to promote glucose transport (12, 13), phosphorylating enzymes such as ACC1 to inhibit fatty acid synthesis, and regulating transcription of genes, such as glucose-6-phosphatase (G6PC), phosphoenolpyruvate carboxykinase (PCK1), fatty acid synthase (FASN) and ACC1, to inhibit gluconeogenesis and fatty acid synthesis (14, 15, 16). Decreased AMPK activity is observed in human diseases with insulin resistance, *e.g.*, diabetes mellitus and the metabolic syndrome (17).

IQGAP1 is a scaffold that regulates diverse processes in numerous cells and tissues by interacting with over 100 binding partners, including several protein kinases (18). IQGAP1 scaffolds components of certain intracellular signaling pathways to facilitate signaling. For example, IQGAP1 modulates mitogen-activated protein kinase (MAPK) signaling by directly binding to B-Raf, MAPK/ERK kinase (MEK), and extracellular signal-regulated kinase (ERK) to facilitate ERK phosphorylation (19, 20, 21). Similarly, in the phosphoinositide-3-kinase (PI3K) pathway, IQGAP1 assembles multiple components of the PI3K signaling cascade to facilitate AKT signaling (22, 23, 24). In addition, Ca^2+^ regulates IQGAP1 *via* CaM. CaM binds to the IQ domain of IQGAP1, which alters numerous interactions of IQGAP1, such as its binding to B-Raf (25), epidermal growth factor receptor (26), CDC42 (27, 28), E-cadherin (29), and RAP1 (30).

IQGAP1 regulates several biological processes, and recent studies have demonstrated roles for IQGAP1 in metabolic pathways. For example, IQGAP1 binds directly to the insulin receptor and associated downstream components to facilitate insulin signaling (31). Importantly, both insulin signaling and glucose homeostasis are impaired in IQGAP1-null mice. Consistent with our observations in mice, IQGAP1 expression is reduced in adipocytes derived from patients with type 2 diabetes mellitus (32). In addition, IQGAP1-null mice display defects in the fasting response, with impaired ketogenesis and reduced fatty acid oxidation (33). Since AMPK is also involved in insulin resistance and metabolic signaling (17), we sought to determine if IQGAP1 modulates AMPK signaling.

Here we identified direct interactions between IQGAP1 and both AMPK and CaMKK2. Reduction of IQGAP1 expression resulted in diminished AMPK activation in several cell types. In addition, reduced IQGAP1 expression decreased Ca^2+^-stimulated AMPK activation. Further, loss of IQGAP1 from mice impaired transcription of gluconeogenesis and fatty acid synthesis genes.

## Results

### IQGAP1 interacts with the α1 subunit of AMPK

**Figure 1:**
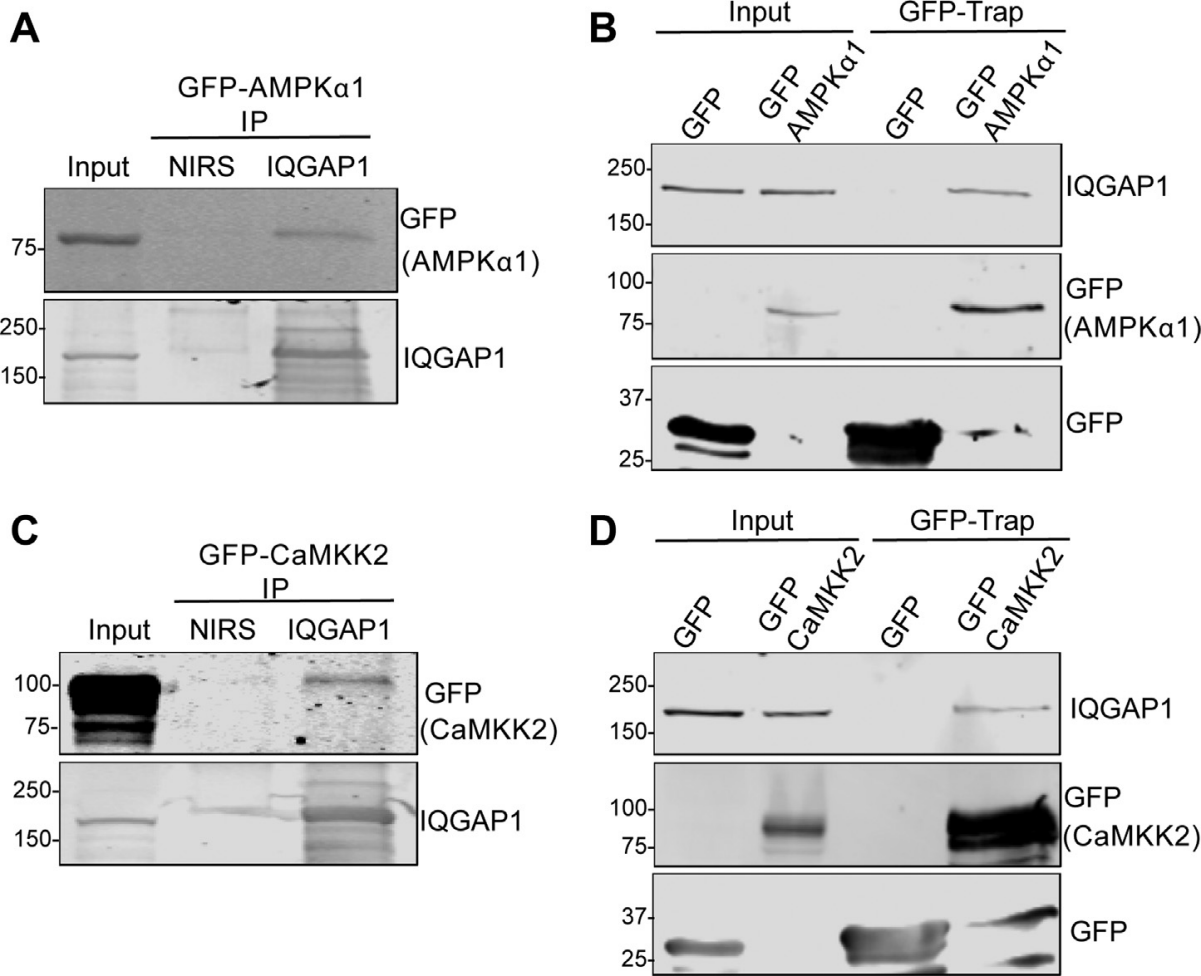
**AMPKα1 and CaMKK2 co-immunoprecipitate with IQGAP1.***A*, HEK-293 cells were transfected with GFP-AMPKα1. 48 h after transfection, cells were lysed and endogenous IQGAP1 was immunoprecipitated (IP) with anti-IQGAP1 antibody (IQ1). Nonimmune rabbit serum (NIRS) was used as control. Samples were analyzed by SDS-PAGE and Western blotting using anti-GFP and anti-IQGAP1 antibodies. 1% of the lysate used for IP was resolved in parallel (Input). *B*, HEK-293 cells were transfected with GFP or GFP-AMPKα1. 72 h after transfection, cells were lysed and GFP-tagged proteins were immunoprecipitated with GFP-Trap Agarose. Samples were analyzed by SDS-PAGE and Western blotting using anti-GFP and anti-IQGAP1 antibodies. 1% of the lysate used for IP was resolved in parallel (Input). *C*, HEK-293 cells were transfected with GFP-CaMKK2. Immunoprecipitation of endogenous IQGAP1 and Western blotting were performed as described for panel (*A*). *D*, HEK-293 cells were transfected with GFP or GFP-CaMKK2. Immunoprecipitation of GFP proteins and Western blotting were performed as in panel (*B*). The positions of migration of molecular weight markers are indicated on the left. All data are representative of at least three independent experiments.

We previously documented that IQGAP1 binds directly to components of the MAPK (19, 20, 21) and the PI3K (23) signaling cascades. To ascertain if IQGAP1 can interact with AMPK, we transfected HEK293 cells with a GFP-tagged construct of the α1 subunit of AMPK. IQGAP1 was immunoprecipitated from cell lysates, and samples were resolved by SDS-PAGE. Western blotting revealed that AMPKα1 bound to endogenous IQGAP1 (Fig. 1*A*). Nonimmune rabbit serum (NIRS) was used as a negative control. AMPKα1 was not seen in the NIRS samples, verifying that the interaction between IQGAP1 and AMPKα1 is specific.

To confirm the interaction, the reverse immunoprecipitation was performed. HEK293 cells were transfected with GFP or GFP-AMPKα1. GFP-tagged proteins were immunoprecipitated from cell lysates with GFP-trap Agarose, and samples were resolved by SDS-PAGE. Western blotting revealed that endogenous IQGAP1 bound to GFP-AMPKα1, but not to GFP alone (Fig. 1*B*). These data demonstrate that IQGAP1 and AMPK α1 interact in cells.

### CaMKK2 and IQGAP1 coimmunoprecipitate from cell lysates

To determine whether IQGAP1 associates with other proteins that participate in the AMPK signaling pathway, we examined whether it binds CaMKK2, a kinase that phosphorylates and activates AMPK. We immunoprecipitated IQGAP1 from HEK293 cells, which had been transfected with GFP-CaMKK2. Analogous to our observations with AMPK, CaMKK2 coimmunoprecipitated with endogenous IQGAP1 (Fig. 1*C*). No CaMKK2 coprecipitated with NIRS.

To validate the observation, we performed the reverse immunoprecipitation. We transfected HEK293 cells with GFP or GFP-CaMKK2 and isolated GFP-tagged proteins from cell lysates with GFP-trap Agarose. Western blotting showed that endogenous IQGAP1 bound to GFP-CaMKK2, but not to GFP alone (Fig. 1*D*). Together these data strongly suggest that IQGAP1 associates with both AMPKα1 and CaMKK2 in cells.

### AMPKα1 binds directly to the IQ domain of IQGAP1

**Figure 2:**
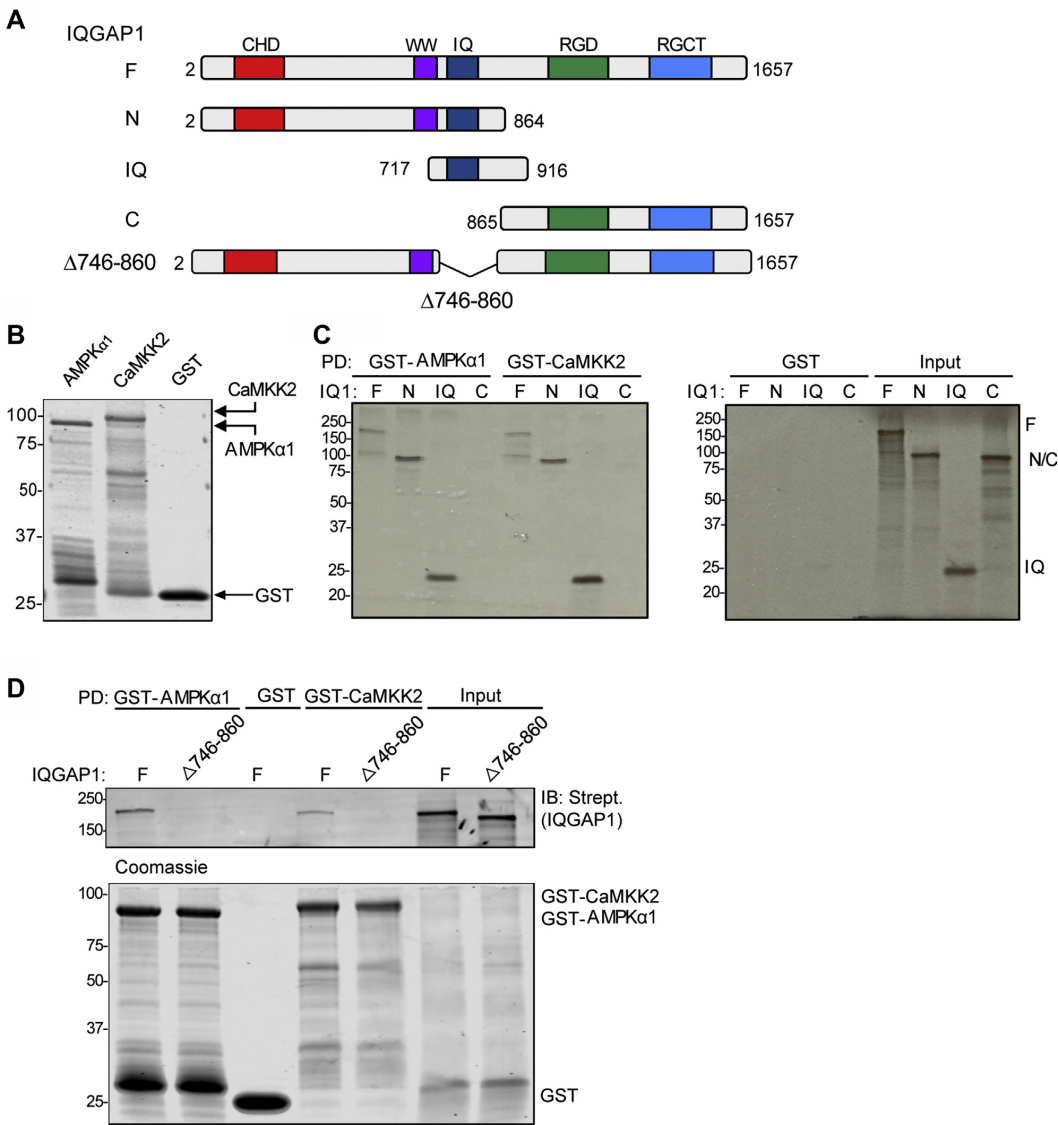
**AMPKα1 and CaMKK2 bind directly to IQGAP1 *via* its IQ domain.***A*, schematic representation of IQGAP1 constructs. The identified protein interaction motifs (*CHD*, calponin homology domain; *WW*, two tryptophan-containing domain; *IQ*, IQ domain; *GRD*, GAP-related domain; *RGCT*, RasGAP C_terminus) and amino acid residues of each construct are indicated. These constructs correspond to full-length IQGAP1 (F, amino acids 2–1657), the N-half (N, 2–863), the IQ domain (IQ, 717–916), and the C-half (C, 864–1657) of IQGAP1. IQGAP1Δ746-860 has amino acids 746 to 860 deleted. *B*, a Coomassie-stained gel of the GST proteins (GST-AMPKα1, GST-CaMKK2, or GST alone) used for binding assays. Data are representative of two independent experiments. *C*, fragments of IQGAP1 (F, N, IQ or C) were expressed and labeled with [^35^S]methionine using the T_N_T system. The IQGAP1 (IQ1) fragments were incubated with purified recombinant GST-AMPKα1, GST-CaMKK2, or GST alone. Complexes were pulled down (PD) with glutathione-Sepharose beads and analyzed by SDS-PAGE and autoradiography. 1% of the T_N_T products were analyzed in parallel (Input, right panel). The positions of migration of molecular weight markers are indicated on the left. Data are representative of at least two independent experiments. *D*, T_N_T products of full-length IQGAP1(F) or IQGAP1Δ746-860 (Δ746-860) were labeled with biotinylated-lysine and were incubated with purified GST alone, GST-AMPKα1, or GST-CaMKK2. Complexes were pulled down and analyzed by SDS-PAGE. The gel was cut at ∼120 kDa. The upper portion of the gel (containing IQGAP1) was processed by Western blotting using IRDye-conjugated streptavidin (Strept.). The lower portion of the gel was stained with Coomassie blue. 1% of the T_N_T products were analyzed in parallel (Input). Data are representative of five independent experiments.

To assess if IQGAP1 binds directly to AMPKα1, we performed *in vitro* binding assays with pure proteins. Full-length IQGAP1 and selected portions of IQGAP1 (Fig. 2*A*) were expressed by *in vitro* transcription and translation (T_N_T) and labeled with [^35^S]methionine in a reticulocyte lysate. Recombinant GST-tagged AMPKα1 was generated, expressed in *Escherichia coli* and purified (Fig. 2*B*). The [^35^S]methionine-labeled constructs were incubated with GST-AMPKα1, and the fragments that bound were resolved by SDS-PAGE. Analysis by autoradiography showed that full-length IQGAP1 bound to GST-AMPKα1 (Fig. 2*C*, left panel). Examination of the two halves of IQGAP1 revealed that the N-terminal half (amino acids 2–863), but not the C-half of IQGAP1, bound to AMPKα1 (Fig. 2*C*, left panel). We previously observed that several proteins interact with the IQ domain of IQGAP1 (18). Therefore, we examined whether this region is sufficient to bind AMPKα1. Pull-down with GST-AMPKα1 demonstrated a clear interaction with the IQ peptide (amino acids 717–916) (Fig. 2*C*, left panel). None of the IQGAP1 constructs bound to GST alone (Fig. 2*C*, right panel), validating the specificity of binding to AMPKα1.

### CaMKK2 binds directly to the IQ domain of IQGAP1

Similar binding analysis was performed with GST-CaMKK2. Equal amounts of each of the IQGAP1 fragments (Fig. 2*C*, input) were incubated with the GST-CaMKK2 construct. Analogous to the observations with AMPKα1, full-length IQGAP1 bound CaMKK2 *via* its N-terminal half; the C-terminal fragment did not bind (Fig. 2*C*, left panel). Moreover, the IQ region was the smallest fragment of IQGAP1 that bound. Taken together, these results reveal that AMPKα1 and CaMKK2 bind directly to IQGAP1 *via* its IQ domain.

If the IQ domain of IQGAP1 is required for its interaction with AMPKα1 or CaMKK2, deletion of this region should abrogate binding. To test this hypothesis, we used an IQGAP1 construct, termed IQGAP1Δ746-860, which lacks the IQ domain (Fig. 2*A*) (19). IQGAP1Δ746-860 and full-length IQGAP1, expressed and labeled with biotinylated-lysine by T_N_T, were incubated with purified GST-tagged AMPKα1. Samples were resolved by SDS-PAGE, and Western blots were probed with labeled streptavidin, which has a high affinity for biotin. As was observed with the [^35^S]methionine-labeled construct, full-length IQGAP1 bound to GST-AMPKα1 (Fig. 2*D*, left panel). By contrast, no IQGAP1Δ746-860 was detected in the GST-AMPKα1 pulldown. Analogous experiments were performed to evaluate binding to GST-CaMKK2. The results were essentially the same as we observed with GST-AMPKα1; while biotinylated, full-length IQGAP1 readily formed a complex with CaMKK2, IQGAP1Δ746-860 was unable to bind (Fig. 2*D*). No IQGAP1 protein was detected in pulldowns with GST alone. The lower panels in Figure 2*D* are Coomassie-stained gels, which demonstrate that equal amounts of GST-AMPKα1 or GST-CaMKK2 were incubated with each IQGAP1 protein. Moreover, equal amounts of each of the biotinylated IQGAP1 proteins were incubated with each GST construct (Fig. 2*D*, Input). These results reveal that the IQ domain of IQGAP1 is both necessary and sufficient for its interaction with either AMPKα1 or CaMKK2.

### Loss of IQGAP1 impairs activation of AMPK by Ca^2+^

**Figure 3:**
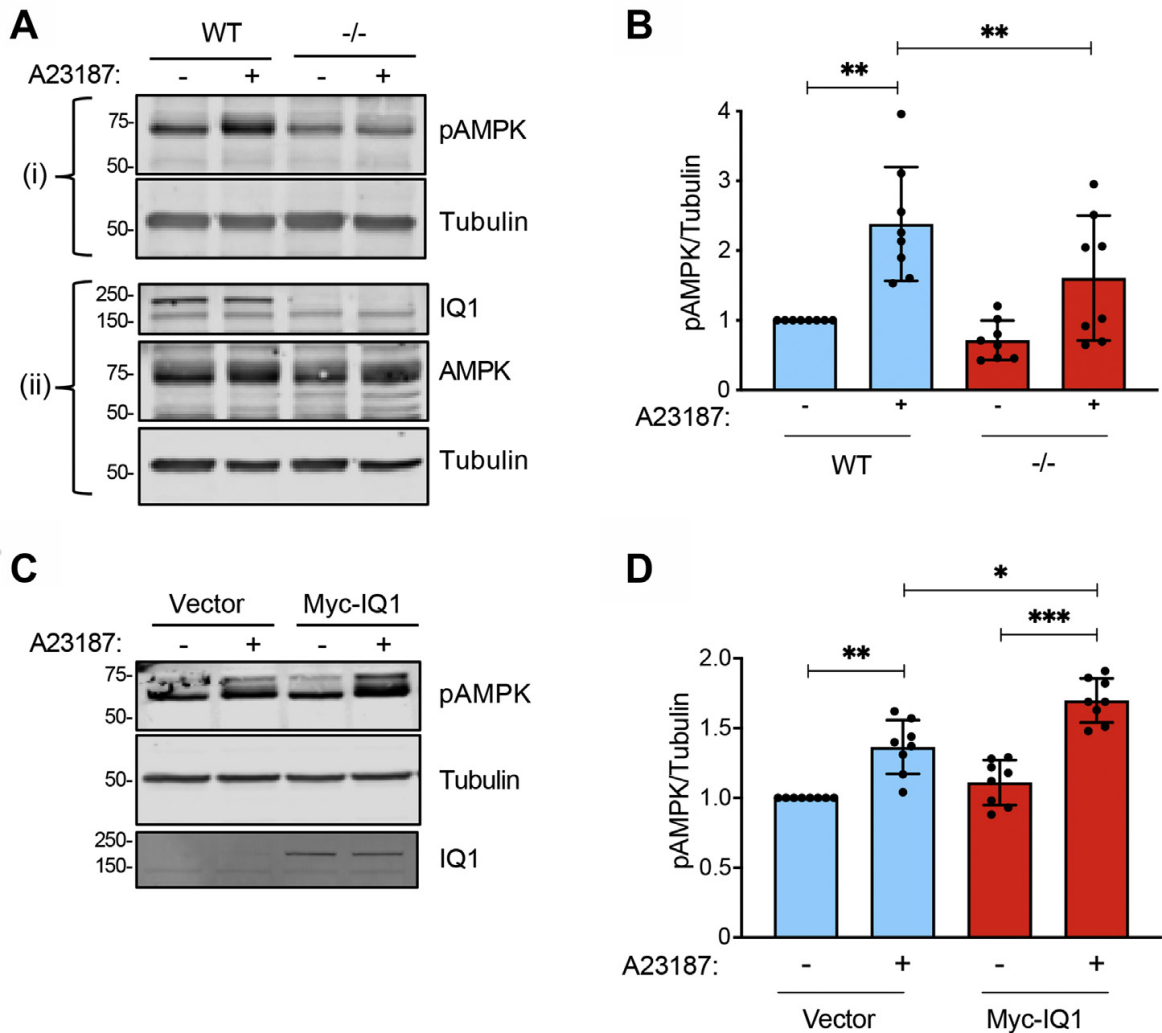
**IQGAP1 influences AMPK activation by Ca**^**2+**^**.***A*, wild-type (WT) and IQGAP1-null (−/−) MEF cells were treated with DMSO (−) or 10 μM A23187 (+) for 15 min. Cells were harvested and equal amounts of protein lysate were analyzed by Western blotting. Two separate blots (i and ii) were performed from the same lysates to probe for multiple proteins. Blot (i) was probed using anti-pAMPK and antitubulin (loading control) antibodies. Blot (ii) was probed with anti-IQGAP1 (IQ1), anti-AMPK, and antitubulin antibodies. The positions of migration of molecular weight markers are indicated on the left. *B*, the pAMPK and tubulin bands from the same blots were quantified with Image Studio 2.0 and pAMPK was corrected for the amount of tubulin in the corresponding sample. Data are expressed as mean ± SD (*n* = 8) with vehicle-treated wild-type cells set as 1. *C*, IQGAP1-null MEF cells were transfected with empty vector or Myc-IQGAP1 (Myc-IQ1). 48 h after transfection, cells were treated with DMSO (−) or 10 μM A23187 (+) for 15 min. Cells were harvested and equal amounts of protein lysate were analyzed by Western blotting using anti-pAMPK, anti-IQGAP1, and antitubulin antibodies. *D*, the pAMPK/tubulin ratios were calculated as described in panel *B*. Data are expressed as mean ± SD (*n* = 8) with vehicle-treated cells transfected with empty vector set as 1. All statistical analyses were performed using one-way ANOVA, with Tukey’s post-hoc test. ∗*p* < 0.05; ∗∗*p* < 0.01; ∗∗∗*p* < 0.001.

To evaluate if IQGAP1 influences activation of AMPK by CaMKK2, we used A23187. The ionophore A23187 increases [Ca^2+^]_*i*_, thereby activating AMPK *via* CaMKK2. We compared the effect of A23187 on mouse embryonic fibroblasts (MEFs) from IQGAP1-null mice and littermate controls. MEFs were incubated with A23187 or DMSO (vehicle control), and phosphorylation of AMPK was evaluated by Western blotting. A23187 stimulated a 2.38 ± 0.82-fold (mean ± SD) increase in AMPK phosphorylation in control MEFs (Fig. 3, *A*–*B*). While A23187 also enhanced AMPK phosphorylation in IQGAP1-null MEFs, the amount of active AMPK was significantly less than that in control cells. To determine if reexpression of IQGAP1 can rescue Ca^2+^-mediated stimulation of AMPK phosphorylation, IQGAP1-null MEFs were transfected with Myc-IQGAP1. A23187 enhanced AMPK phosphorylation by 1.37 ± 0.19-fold (mean ± SD) in cells transfected with empty vector. In cells reconstituted with IQGAP1, the stimulation of AMPK phosphorylation induced by [Ca^2+^]_*i*_ was significantly greater than that in IQGAP1-null MEFs (Fig. 3, *C*–*D*). Importantly, due to its large size, IQGAP1 is expressed in only 22% of the rescue MEFs (data not shown). These observations suggest that the effect of IQGAP1 is even more robust than indicated in the figures. Taken together, these data demonstrate that IQGAP1 is required for maximal activation of AMPK in MEFs by increased [Ca^2+^]_*i*_.

### Knockdown of IQGAP1 reduces AMPK activation in human cell lines

**Figure 4:**
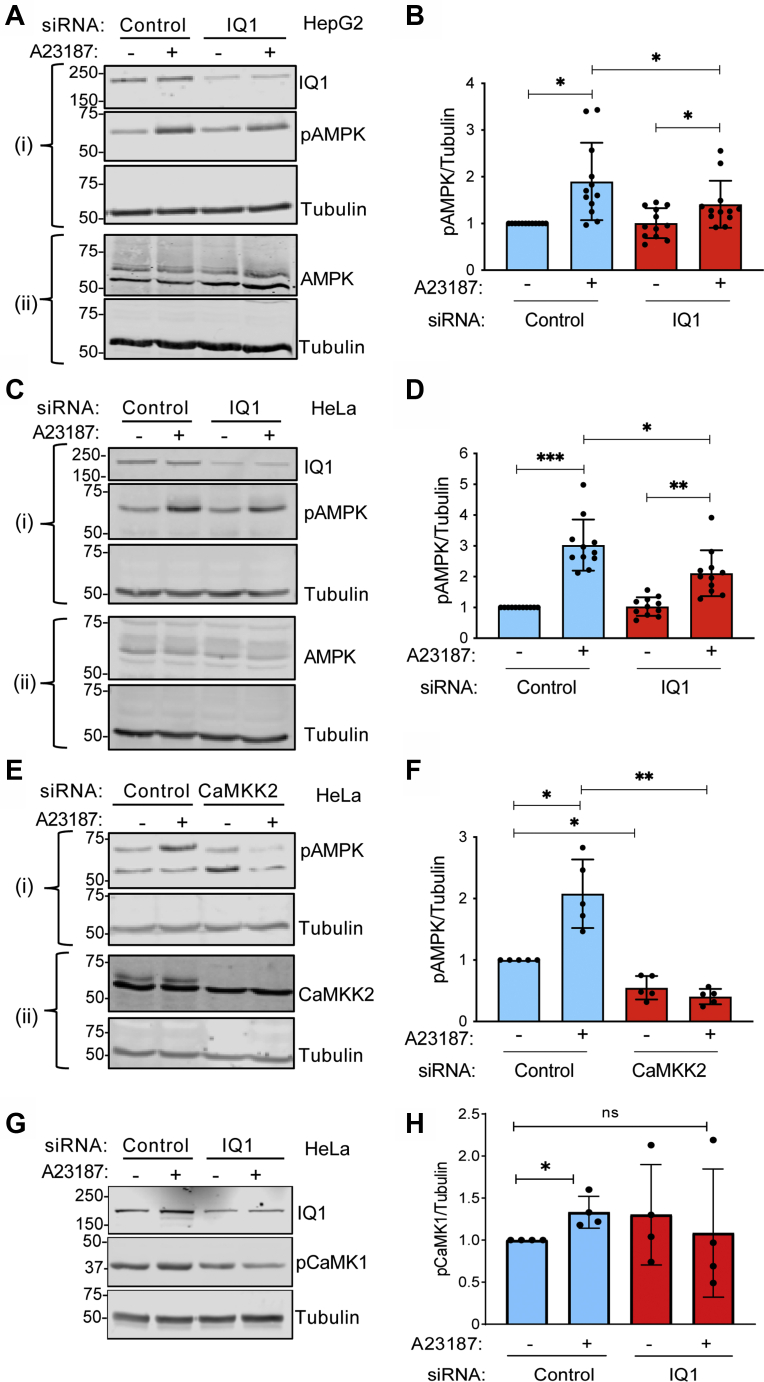
**Knockdown of IQGAP1 alters AMPK activation in human cell lines.***A*, HepG2 cells were transfected with control or IQGAP1 (IQ1)-targeted siRNA. 72 h after transfection, DMSO (−) or 10 μM A23187 (+) was added for 15 min. Cells were harvested and equal amounts of protein lysate were analyzed by Western blotting. Two separate blots (i and ii) were performed to probe for multiple proteins. Blot (i) was probed with anti-IQGAP1, anti-pAMPK, and antitubulin (loading control) antibodies. Blot (ii) was probed with anti-AMPK and antitubulin antibodies. *B*, the pAMPK and tubulin bands from the same blots were quantified with Image Studio 2.0 and corrected for the amount of tubulin in the corresponding sample. Data are expressed as mean ± SD (*n* = 12) with vehicle-treated cells transfected with control siRNA set as 1. *C*, HeLa cells were transfected with control or IQGAP1-targeted siRNA. 72 h after transfection, cells were treated with DMSO (−) or 10 μM A23187 (+) for 15 min. Samples were processed as described for panel A. *D*, the pAMPK/tubulin ratios were calculated as described in panel B. Data are expressed as mean ± SD (*n* = 11) with vehicle-treated cells transfected with control siRNA set as 1. *E*, HeLa cells were transfected with control or CaMKK2-targeted siRNA. 72 h after transfection, DMSO (−) or 10 μM A23187 (+) was added for 15 min. Samples were processed as described for panel A. *F*, pAMPK/tubulin ratios were calculated as described for panel B. Data are expressed as mean ± SD (*n* = 5) with vehicle-treated cells transfected with control siRNA set as 1. *G*, equal amounts of protein lysate from the HeLa cells transfected as described in panel C were analyzed by Western blotting. Blots were probed with anti-IQGAP1 (IQ1), anti-pCaMK1, and antitubulin antibodies. *H*, the pCaMK1 and tubulin bands from the same blots were quantified and corrected for the total amount of tubulin in the sample. Data are expressed as mean ± SD (*n* = 4) with vehicle-treated cells transfected with control siRNA set as 1. All statistical analyses were performed using one-way ANOVA, with Tukey’s post-hoc test. ∗*p* < 0.05; ∗∗*p* < 0.01; ∗∗∗*p* < 0.001; ns, not significant.

To determine if IQGAP1 participates in Ca^2+^-stimulated AMPK signaling in human cells, we knocked down IQGAP1 using siRNA in HepG2 cells. Analogous to our observations in MEFs, A23187 stimulated a significant (1.90 ± 0.83-fold) increase in AMPK phosphorylation in HepG2 cells transfected with control siRNA (Fig. 4, *A*–*B*). The ability of increasing [Ca^2+^]_*i*_ to activate AMPK was significantly attenuated when IQGAP1 was knocked down in HepG2 cells. Basal AMPK phosphorylation was unchanged by IQGAP1 knockdown (Fig. 4, *A*–*B*).

To further evaluate the role of IQGAP1 in AMPK activation by CaMKK2, we selected HeLa cells. Because the HeLa cell line lacks LKB1 (4), AMPK phosphorylation is mediated primarily by CaMKK2. In cells transfected with control siRNA, A23187 stimulated a 3.03 ± 0.83-fold (mean ± SD) increase in AMPK activation (Fig. 4, *C*–*D*). By contrast, when IQGAP1 is knocked down, the increase in AMPK phosphorylation induced by A23187 is significantly attenuated. As observed with HepG2 cells, reduction of IQGAP1 by siRNA in HeLa cells did not alter basal pAMPK (Fig. 4, *C*–*D*). These data validate that IQGAP1 is required for CaMKK2 to maximally activate AMPK.

In order to confirm that the effects we observed in HeLa cells are mediated *via* CaMKK2, we repeated the analyses in cells in which CaMKK2 was knocked down by siRNA. Basal AMPK phosphorylation was significantly reduced in HeLa cells transfected with CaMKK2 siRNA (Fig. 4, *E*–*F*). Moreover, A23187 was unable to enhance AMPK activation in cells with CaMKK2 knockdown. These data reveal that A23187 stimulates CaMKK2 to activate AMPK in HeLa cells.

### Knockdown of IQGAP1 reduces Ca^2+^-stimulated CaMKK2 activity

We examined the effect of IQGAP1 knockdown on CaMKK2 actvity by evaluating its ability to phosphorylate CaMK1. CaMK1 phosphorylation on Thr-177 was examined in HeLa cells transfected with control siRNA or IQGAP1-targeted siRNA. In cells transfected with control siRNA, A23187 significantly increased CaMK1 phosphorylation (4G and H). By contrast, when IQGAP1 was knocked down, Ca^2+^ was unable to stimulate CaMK1 phosphorylation. These data show that Ca^2+^-stimulated CaMKK2 activity is decreased when IQGAP1 protein levels are reduced.

### Effect of knockdown and reconstitution of IQGAP1 in HeLa cells on activation of AMPK by Ca^2+^

**Figure 5:**
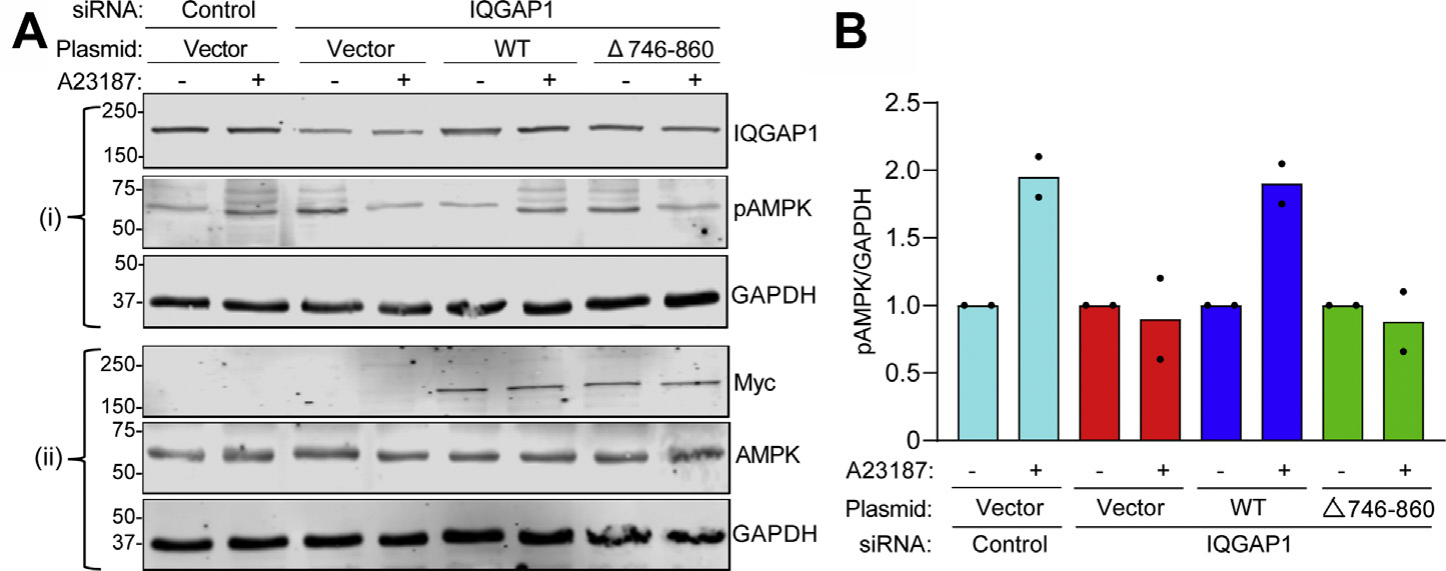
**IQGAP1Δ746-860 fails to rescue AMPK activation by Ca**^**2+**^**in cells with reduced endogenous IQGAP1.***A*, to knockdown endogenous IQGAP1, HeLa cells were transfected with IQGAP1-targeted siRNA (siIQGAP1-8). Scrambled siRNA was used as the control. 24 h later the cells were transfected with the following plasmids: empty vector or Myc-tagged constructs of wild-type IQGAP1 (WT) or IQGAP1Δ746-860 (Δ746-860). 48 h after transfection, cells were treated with DMSO (−) or 10 μM A23187 (+) for 15 min. Cells were harvested and equal amounts of protein lysate were analyzed by Western blotting. Two separate blots (i and ii) were performed to probe for multiple proteins. Blot (i) was probed with anti-IQGAP1, anti-pAMPK, and anti-GAPDH (loading control) antibodies. Blot (ii) was probed with anti-Myc, anti-AMPK, and anti-GAPDH antibodies. *B*, the pAMPK and GAPDH bands from the same blots were quantified by densitometry using Image Studio 2.0 and pAMPK was corrected for the amount of GAPDH in the corresponding sample. Data are expressed as means (n = 2) with vehicle-treated cells in each condition set as 1.

We also used a second siRNA to evaluate the effect of IQGAP1 knockdown on AMPK activation by Ca^2+^. Endogenous IQGAP1 expression was reduced in HeLa cells using a single IQGAP1 siRNA oligonucleotide. We previously documented that this siRNA reduced IQGAP1 protein expression by ∼80% (34). In cells transfected with a scrambled control siRNA sequence, A23187 increased pAMPK by 1.95-fold (Fig. 5, *A*–*B*). Consistent with our earlier data with a pooled siRNA (Fig. 4, *C*–*D*), knockdown of IQGAP1 with siIQGAP1-8 resulted in markedly decreased AMPK activation by Ca^2+^ (Fig. 5, *A*–*B*).

To determine if reexpression of IQGAP1 can rescue Ca^2+^-mediated AMPK activation in HeLa cells, we transfected the IQGAP1 knockdown cells with siRNA resistant wild-type IQGAP1. The level of IQGAP1 expression in the reconstituted cells was essentially the same as that in control cells (Fig. 5*A*). A23187 increased AMPK phosphorylation by 1.9-fold in cells reconstituted with wild-type IQGAP1, which was the same magnitude as that observed in control cells (without IQGAP1 knockdown) (Fig. 5, *A*–*B*).

If the interaction between IQGAP1 and AMPK and/or CaMKK2 is required for Ca^2+^ to activate AMPK, one would anticipate that an IQGAP1 construct that is unable to bind AMPK and CaMKK2 would not be able to rescue the effect of IQGAP1 knockdown. Therefore, we reconstituted IQGAP1 knockdown HeLa cells with siRNA resistant IQGAP1Δ746-860. Importantly, A23187 was unable to increase AMPK activation in cells expressing IQGAP1Δ746-860 (Fig. 5, *A*–*B*). Note that the amount of IQGAP1Δ746-860 protein expressed was the same as that in control cells and in cells reconstituted with wild-type IQGAP1. Taken together, these data demonstrate that reexpression of wild-type IQGAP1 can rescue siRNA-mediated defects in AMPK activation and that an interaction of AMPKα1 and/or CaMKK2 with IQGAP1 is required for maximal Ca^2+^-mediated AMPK phosphorylation.

### Absence of IQGAP1 abrogates AMPK activation by metformin

**Figure 6:**
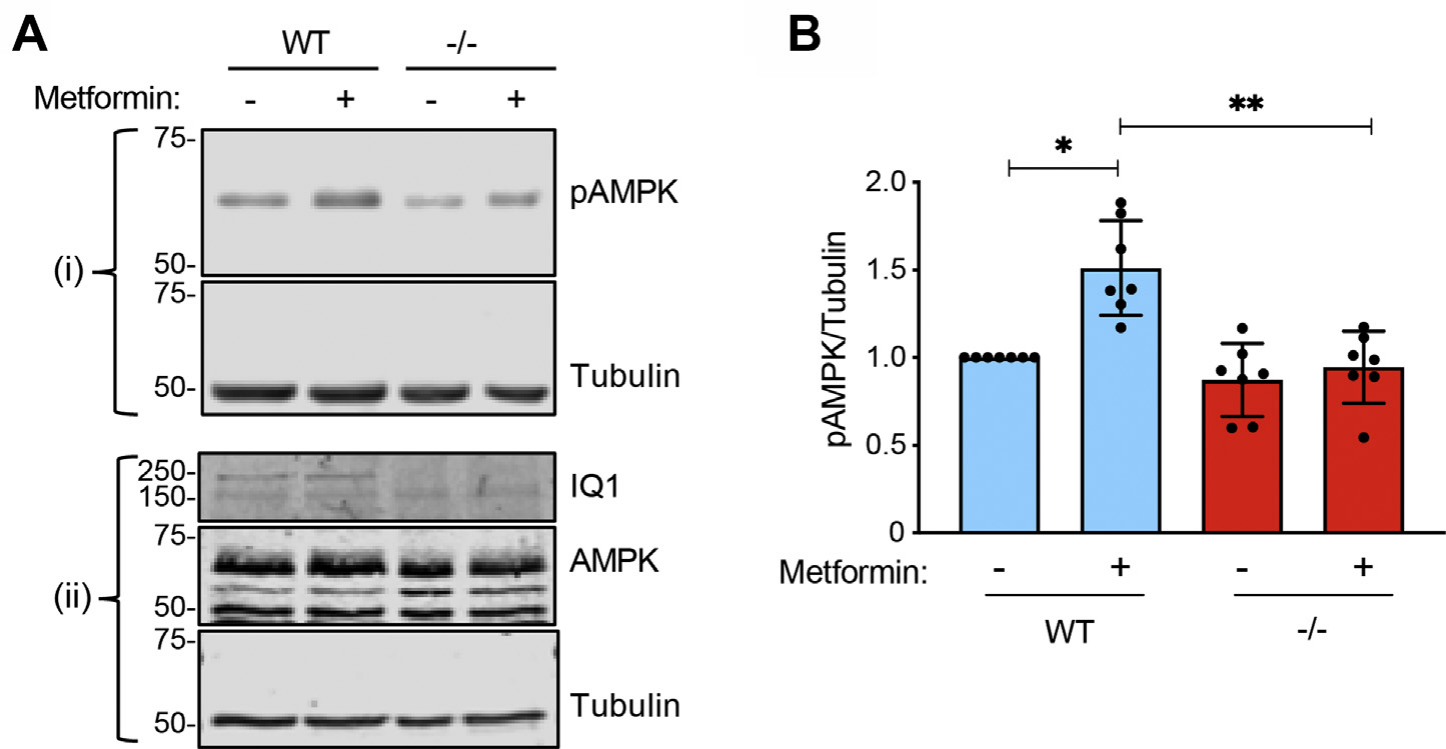
**IQGAP1 influences AMPK activation by metformin.***A*, wild-type (WT) and IQGAP1-null (−/−) MEF cells were treated without (−) or with (+) 5 mM metformin for 4 h. Cells were harvested and equal amounts of protein lysate were analyzed by Western blotting. Two separate blots (i and ii) were performed from the same lysates to probe for multiple proteins. Blot (i) was probed using anti-pAMPK and antitubulin (loading control) antibodies. Blot (ii) was probed with anti-IQGAP1 (IQ1), anti-AMPK, and antitubulin antibodies. The positions of migration of molecular weight markers are indicated on the left. *B*, the pAMPK and tubulin bands from the same blots were quantified with Image Studio 2.0 and pAMPK was corrected for the amount of tubulin in the corresponding sample. Data are expressed as mean ± SD (*n* = 7) with untreated wild-type cells set as 1. Statistical analysis was performed using one-way ANOVA, with Tukey’s post-hoc test. ∗*p* < 0.05; ∗∗*p* < 0.01.

To determine whether IQGAP1 modulates AMPK signaling independently of CaMKK2, we used metformin. Metformin activates AMPK *via* LKB1 (35) by altering mitochondrial respiration and cellular AMP and ADP levels (13). In MEFs obtained from wild-type mice, metformin induced a significant increase (1.51- ± 0.27-fold; mean ± SD) in AMPK phosphorylation (Fig. 6, *A*–*B*). By contrast, metformin was unable to enhance AMPK phosphorylation in IQGAP1-null cells. The absence of IQGAP1 did not significantly alter AMPK phosphorylation in untreated cells (Fig. 6, *A*–*B*). These data indicate that IQGAP1 is required for AMPK activation by metformin in mouse fibroblasts.

### IQGAP1-null mice displayed altered fasting response

**Figure 7:**
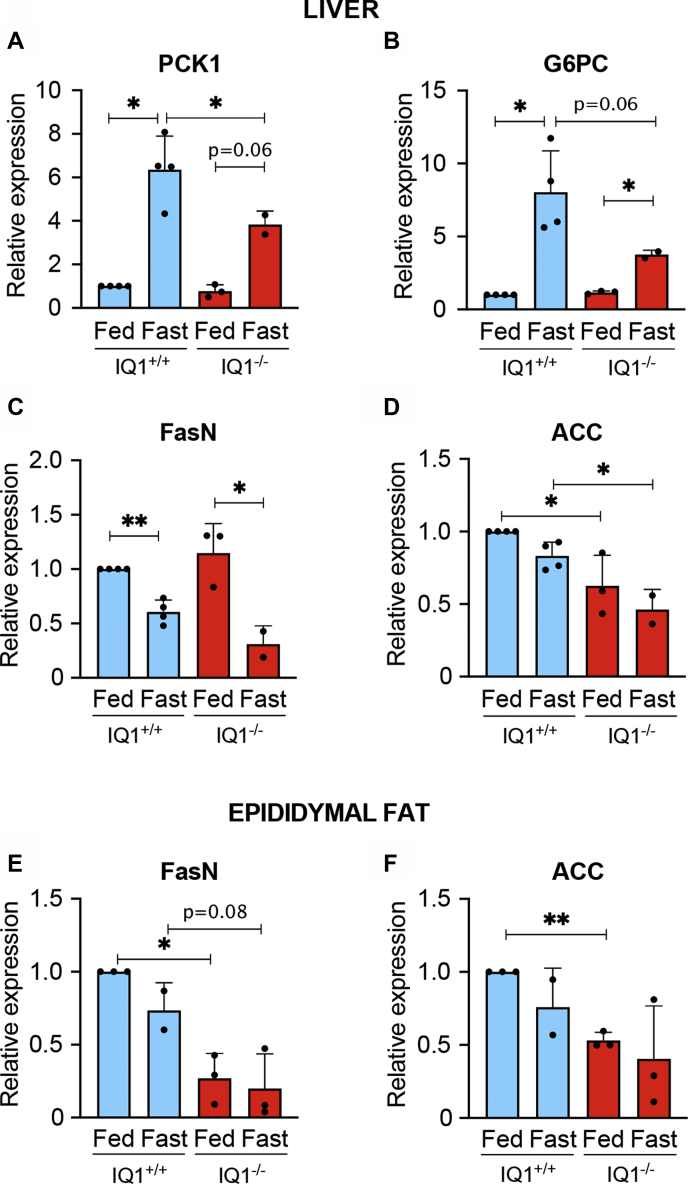
**Expression of gluconeogenesis and fatty acid synthesis genes in IQGAP1-null mice.** Wild-type (IQ1^+/+^) or IQGAP1-null (IQ1^−/−^) mice were fed or fasted for 16 h. Then, mice were euthanized with carbon dioxide and tissues were extracted. Quantitative RT-PCR analysis was performed to measure mRNA expression of PCK1 and G6PC in the liver (*panels A* and *B*) and FASN and ACC1 in the liver (*panelsC* and *D*) and epididymal fat pads (*panels E* and *F*). Expression of genes was normalized to the housekeeping gene 18S RNA. Data are expressed as mean ± SD (n = 2–4 mice per group, each assay was performed in triplicate) with fed wild-type mice set as 1. Statistical analysis was performed using unpaired *t*-test with Welch’s correction. ∗*p* < 0.05; ∗∗*p* < 0.01.

To assess the possible role of IQGAP1 in the response to nutrient deprivation *in vivo*, we used IQGAP1-null mice (36). One group of mice was maintained on a normal diet, while the other group was fasted for 16 h with water provided *ad libitum*. RNA was purified from both the liver and epididymal fat and changes in gene expression between wild-type and IQGAP1-null mice under fed or fasted conditions were examined by quantitative RT-PCR. We analyzed hepatic mRNA expression of two gluconeogenic genes, namely PCK1 and G6PC, which are induced by fasting (37). As expected, both PCK1 and G6PC mRNA levels increased significantly in fasted wild-type mice (Fig. 7, *A*–*B*). The expression of PCK1 and G6PC was 5.4- and 4.4-fold, respectively, higher in fasted wild-type mice than in fed mice. Expression of PCK1 and G6PC in fed IQGAP1-null mice was essentially the same as in fed wild-type mice (Fig. 7, *A*–*B*). By contrast, when IQGAP1-null mice were fasted, the magnitude of the increased expression of PCK1 and G6PC was less than that in wild-type mice.

The effect of IQGAP1 on AMPK-regulated fatty acid synthesis was also evaluated. We analyzed mRNA expression of FASN and ACC1, two genes that promote fatty acid synthesis and are regulated by AMPK signaling (14, 15, 16) (Fig. 7, *C*–*F*). When control mice were fasted, FASN expression decreased significantly in the liver and fat (Fig. 7, *C*–*E*). FASN expression in the liver of fed wild-type and IQGAP1-null mice was similar. However, in fat it was significantly (reduction of 73%) lower in fed IQGAP1-null mice than in fed control mice. Interestingly, fasting significantly decreased FASN only in the liver of IQGAP1-null mice; no alteration was observed in epididymal fat (Fig. 7, *C*–*E*). Analysis of ACC1 in wild-type mice revealed that fasting attenuated mRNA expression in the liver and fat, but the magnitude was <25% and did not reach statistical significance (Fig. 7, *D*–*F*). The response of IQGAP1-null mice to fasting was essentially the same. Interestingly, IQGAP1-null mice had lower ACC1 expression than wild-type mice (Fig. 7, *D*–*F*). This decrease was significant in the liver, being 37% and 55% lower in fed and fasted mice, respectively.

## Discussion

Initially, IQGAP1 was thought to regulate only the cytoskeleton (38). Subsequent work revealed that IQGAP1 is a scaffold that participates in multiple signaling cascades, such as the MAPK (21) and PI3K/Akt (23) signaling pathways. More recent evidence has shown that IQGAP1 also modulates metabolic processes, including those controlled by insulin (31), which has a critical role in glucose homeostasis and lipid metabolism (39), and the nutrient sensor mTor (33), which coordinates lipogenesis (40). By interacting with and assembling selected proteins and molecules, IQGAP1 integrates signaling pathways modulating many cellular functions (18). In this study, we demonstrate a previously unidentified role for IQGAP1 as a component of AMPK signaling.

Here, we demonstrate that IQGAP1 binds directly to the α1 subunit of AMPK. In addition, we identified direct binding of IQGAP1 to CaMKK2, a kinase that catalyzes phosphorylation and activation of AMPK. Interestingly, we observed that both AMPK α1 and CaMKK2 associate with the IQ domain of IQGAP1. This is reminiscent of our prior finding that both the p85 subunit of PI3K and PIPKIα, the kinase immediately upstream of it in the PI3K/AKT signaling cascade, bind to the IQ region of IQGAP1 (23). In addition to the direct *in vitro* binding, we found that IQGAP1 associates with both CaMKK2 and AMPK in cells. The latter finding is consistent with an interactome analysis of AMPK α1 and β1 in pancreatic cells (41). IQGAP1 was one of the 381 proteins identified, but the authors did not investigate potential functional consequences. While IQGAP1 is known to bind directly to CaM (28), to the best of our knowledge, there is no publication that has demonstrated an interaction between CaMKK2 and IQGAP1. Therefore, we designed experiments to explore the potential functional role of IQGAP1 in CaMKK2-mediated activation of AMPK.

AMPK is activated by phosphorylation of Thr-172 in the α subunit, which is catalyzed by either LKB1 or CaMKK2 (2, 3, 4, 5). Diverse extracellular stimuli, such as hormones, adipogenic molecules, and other circulatory factors, bind to their respective receptors to trigger a transient rise in [Ca^2+^]_i,_ enabling formation of Ca^2+^/CaM (42). The Ca^2+^/CaM binds to and activates CaMKK2, which can then phosphorylate and activate AMPK (43, 44). We used several complementary approaches to examine the potential role of IQGAP1 in the activation of AMPK by CaMKK2. We first evaluated the ability of increasing [Ca^2+^]_i_ to activate AMPK in cells lacking IQGAP1. Analysis revealed that Ca^2+^-stimulated phosphorylation of AMPK was significantly lower in IQGAP1-null mouse fibroblasts than in control MEFs. Consistent with the hypothesis that the attenuated response was due to the absence of IQGAP1, we observed that reexpression of IQGAP1 in the cells lacking IQGAP1 restored AMPK activation by A23187 in MEFs lacking IQGAP1. In addition, siRNA-mediated knockdown of IQGAP1 in human cells inhibited the phosphorylation of AMPK induced by Ca^2+^. Moreover, knockdown of IQGAP1 elicited similar results (*i.e.*, impaired AMPK activation by Ca^2+^) in HeLa cells, which lack LKB1 and are thought to depend on CaMKK2 for AMPK activation (2, 3).

To investigate the functional relevance of the association of IQGAP1 with AMPKα1 and CaMKK2, we generated a mutant IQGAP1 construct, termed IQGAP1Δ746-860, that is unable to bind the two kinases. We compared activation of AMPK in HeLa cells (with knockdown of endogenous IQGAP1) reconstituted with wild-type IQGAP1 to cells reconstituted with IQGAP1Δ746-860. While wild-type IQGAP1 completely rescued AMPK phosphorylation, A23187 was unable to stimulate AMPK phosphorylation in cells expressing IQGAP1Δ746-860. These data indicate that the region of IQGAP1 that interacts with AMPKα1 and CaMKK2 is necessary for Ca^2+^ to activate AMPK in cells.

Several prior publications have established that IQGAP1 mediates diverse effects of Ca^2+^, often *via* CaM. The first evidence for this concept was our observation that, in the presence of CaM, Ca^2+^ abrogated the ability of IQGAP1 to regulate CDC42 activity (27, 28). Subsequently, IQGAP1 was shown to integrate Ca^2+^/CaM and B-Raf signaling (19). In addition, Ca^2+^ and cAMP signals are integrated at the leading edges of migrating cells by IQGAP1 in a complex with AKAP220 to control cell migration (45). Moreover, the ability of increased [Ca^2+^]_i_ to induce the release of insulin from the beta cells of the pancreas in response to glucose is impaired by knockdown of IQGAP1 (46). The data presented here show for the first time that IQGAP1 is also required for Ca^2+^ to maximally activate AMPK in cells.

The molecular mechanism(s) by which IQGAP1 modulates AMPK activation is (are) not known. It seems reasonable to postulate that, analogous to its role in the MAPK (19, 20, 21) and PI3K/AKT (23) pathways, IQGAP1 scaffolds AMPK signaling components. In this model, IQGAP1 promotes the coupling between CaMKK2 and AMPKα1 by bringing the two proteins in immediate proximity, thereby facilitating CaMKK2-mediated phosphorylation of AMPK. AMPK function is known to be influenced by scaffold proteins. For example, kinase suppressor of RAS 2 (KSR2), a MAPK scaffold, interacts with the α1 subunit of AMPK, and loss of KSR2 reduces AMPK activation (47). Another scaffold protein, Nischarin, binds both the α subunit of AMPK and LKB1, thereby modulating AMPK signaling (48). A second possibility is that IQGAP1 regulates the activity of CaMKK2. Two mechanisms are feasible. IQGAP1 may act as a source of CaM to activate CaMKK2. IQGAP1 binds both Ca^2+^/CaM and Ca^2+^-free CaM (apoCaM) (49), while CaMKK2 binds to Ca^2+^/CaM) (42). At resting low [Ca^2+^]_i_, apoCaM is constitutively bound to IQGAP1 (28). In response to an extracellular stimulus, a transient increase in [Ca^2+^]_i_ in a localized subcellular region converts the apoCaM bound to IQGAP1 to Ca^2+^/CaM. The high affinity of Ca^2+^/CaM for CaMKK2 induces binding and activation, which leads to AMPK activation. Alternatively, direct binding of IQGAP1 to CaMMK2 may alter the conformation of the enzyme, releasing it from an autoinhibited state and enhancing kinase activity. IQGAP1 is known to augment the activity of other kinases. For example, *in vitro* experiments have demonstrated that IQGAP1 promotes the kinase activity of B-Raf (19) and phosphoinositide kinases (23). The models proposed above are not mutually exclusive, and more than one may operate in cells.

Our data also reveal that IQGAP1 participates in activation of AMPK independently of CaMKK2. We observed that metformin is unable to increase AMPK phosphorylation in IQGAP1-null cells. Metformin is the primary drug used in the treatment of type 2 diabetes (50) and is used by millions of people worldwide. Therefore, a comprehensive understanding of how it elicits its effects is important. Although details of the mechanism by which metformin activates AMPK remain controversial (50), evidence supports a fundamental role for LKB1-catalyzed phosphorylation of AMPK (35). It is not known whether a direct interaction with IQGAP1 is required for AMPK activation by metformin. While we are not aware of any published data that LKB1 binds IQGAP1, it is conceivable that IQGAP1 could act as a scaffold for LKB1 and AMPK, enabling phosphorylation. Additional work is required to elucidate the role of IQGAP1 in AMPK activation by LKB1. Regardless of the mechanism by which IQGAP1 contributes to activation of AMPK by metformin, our collective data suggest that the absence of IQGAP1 leads to a general defect in AMPK activation.

AMPK signaling is critical for the regulation of many aspects of whole-body metabolism. To investigate the potential effects of IQGAP1 on AMPK signaling, we compared the activation of selected genes in IQGAP1-null mice to their littermate controls. We stimulated the AMPK signaling pathway by fasting mice (51, 52, 53). The response of G6PC and PCK1 (which regulate gluconeogenesis) to fasting in the liver of mice lacking IQGAP1 was significantly less than that in wild-type mice. Our findings are very similar to the published observations in hepatocytes derived from the livers of CaMKK2 knockout mice (54). The authors observed that catecholamine-induced gluconeogenesis, assessed by quantifying G6PC and PCK1 mRNA, in hepatocytes was impaired by the absence of CaMKK2. As fasting induces catecholamine release (55), it is possible that the decreased response of G6PC and PCK1 to fasting in IQGAP1-null mice is mediated by CaMKK2. The expression level of the fatty acid synthesis gene FASN in the epididymal fat of IQGAP1-null mice was 73% lower than that in control mice, and the normal decrease in FASN elicited by fasting was absent from the knockout mice. By contrast, fasting reduces the expression of FASN in the livers of both wild-type and IQGAP1-null mice. This response is most likely due to the high levels in the liver of IQGAP2, which has been shown to influence the expression of FASN in mouse liver (56). Since G6PC, PCK1, and FASN are regulated by AMPK signaling and we demonstrate here that IQGAP1 modulates AMPK signaling, we hypothesize that IQGAP1 is involved in fatty acid metabolism and gluconeogenesis at least in part through the AMPK pathway. Notwithstanding our observations, it should be borne in mind that regulation of both gluconeogenesis and fatty acid metabolism *in vivo* involves an intricate interplay of multiple signaling pathways, which are influenced by diverse stimuli from different parts of the body. As mentioned, IQGAP1 participates in several signaling cascades, and some of the effects we observed may be mediated through one or more of these pathways. Regardless of the molecular mechanism, our data strongly suggest that IQGAP1 is necessary for the normal expression and response to fasting of genes that regulate gluconeogenesis and fatty acid synthesis.

Due to its fundamental role in energy control, AMPK has been the focus of intense research over the last few years. A large body of evidence supports the concept that activation of AMPK is beneficial for the prevention and treatment of a variety of chronic diseases, particularly metabolic disorders, such as type 2 diabetes mellitus and the metabolic syndrome (57). We recently documented that IQGAP1 is necessary for optimal activation of insulin signaling, and mice lacking IQGAP1 have impaired glucose tolerance (31), which is a fundamental component of type 2 diabetes. The data presented in this study that IQGAP1 binds AMPK and is required for maximum activation of AMPK raise the possibility that the interaction between AMPK and IQGAP1 could be a potential target for the development of new therapeutic agents for metabolic disorders.

## Experimental procedures

### Materials

Lipofectamine 2000 Reagent (catalogue number 11668019), Lipofectamine LTX Reagent (catalogue number 15338500), and Lipofectamine RNAiMAX (catalogue number 13778150) were obtained from Thermo Fisher Scientific. Glutathione-Sepharose and Protein A-Sepharose beads were purchased from GE Healthcare. GFP-Trap Agarose was obtained from Chromotek (catalogue number gta-20). The pEGFP-C1 and pGEX-2T plasmids were obtained from Clontech and GE Healthcare, respectively. Silencer Negative Control No. 1 siRNA (catalogue number AM4635) and CaMKK2 siRNA (catalogue number AM16708-110916) were obtained from Thermo Fisher Scientific. IQGAP1 siRNA (catalogue number sc-35700) was obtained from Santa Cruz Biotechnology. Custom siRNA sequences for siIQGAP1-8: AAGUUCUACGGGAAGUAA (34) or scrambled: GAUAAAUGGCGCAAUGUA were from Thermo Fisher Scientific.

Metformin was purchased from Selleck Chemicals (catalogue number S1950). A23187 was obtained from Sigma-Aldrich (catalogue number C7522-10MG). TRIzol Reagent was obtained from Invitrogen (catalogue number 15596018). Halt protease inhibitor cocktail was obtained from Thermo Scientific (catalogue number PI78444). Bio-Rad Protein Assay Dye Reagent Concentrate reagent was purchased from Bio-Rad (catalogue number 500-0006). Antibodies and dilutions used are listed in Table 1. Blocking buffer, infrared dye-conjugated (IRDye) secondary antibodies, and IRDye Streptavidin (catalogue number 926-68079) were purchased from Li-COR Biosciences. PVDF membranes were purchased from Millipore Corporation. Unless otherwise stated, all other reagents used were of standard analytical grade.

**Table 1:** Antibodies used in this study

Protein detected	Reference	Dilution for immunoblots
AMPK	Cell Signaling Technology, 2532S	1:1000
Phospho-AMPK T172	Cell Signaling Technology, 2535S	1:1000
β-tubulin	Sigma-Aldrich, T5201-200UL	1:5000
CaMKK2	Sigma-Aldrich, HPA017389-100UL	1:1000
Phospho-CaMK1 T177	Thermo Fisher Scientific, PA538434	1:1000
GAPDH	Cell Signaling Technology, 2118S	1:1000
GFP	Cell Signaling Technology, 2955S	1:1000
IQGAP1	Rabbit serum (27)	1:1000
Myc-Tag, clone 9E10	Millipore Sigma, 05-419	1:1000

### Plasmid construction

pcDNA3-Myc-IQGAP1 (wild-type and Δ746-860 [previously referred to as ΔB-Raf] [19, 29]) plasmids have been previously described. EGFP-C1-AMPKα1 (catalogue number 30305 [58]) and pEGFP-N1-CaMKK2 (catalogue number 32453 [59]) plasmids were purchased from Addgene; both of these GFP-tagged proteins are functional (58, 59). GST-AMPKα1 was made using pEGFP-C1-AMPKα1 as template. The insert was amplified with 5′-GAAGATCTGCCGAGAAGCAGAAGCACGACGGGC-3′ as forward primer and 5′-GCTCTAGACTCGAGTTACTGTGCAAGAATTTTAATTAGAT-3′ as reverse primer. After the plasmid was cut with BglII and XhoI, it was inserted into pGEX4T-TEV (60) at BamHI and XhoI sites. GST-CaMKK2 was made using pEGFP-CaMKK2 as template. The insert was amplified using the 5′-CGGGATCCTCATCATGTGTCTCTAGCCAGCCC-3′ as forward primer and 5′-GCTCTAGATTACAAGAGCACTTCCTCCTCCCCCCAC-3′ as reverse primer. After cutting with BamHI, the plasmid was inserted into pGEX4T-TEV at BamHI and Smal sites. The sequences of all constructs were confirmed by DNA sequencing. All constructs migrated to the expected position on SDS-PAGE.

### Preparation of fusion proteins

GST-IQGAP1 was expressed in *E. coli* and isolated using glutathione-Sepharose essentially as previously described (27). Where indicated, the GST tag was cleaved from GST-IQGAP1 using tobacco etch virus protease as previously described (61). GST-AMPKα1 and GST-CaMKK2 were expressed and purified as described for GST-IQGAP1. Briefly, the recombinant construct pGEX4T-TEV-GST-AMPKα1 was expressed in the BL21 strain of *E. coli* first grown overnight at 25 °C, and expression was induced with 1 μM isopropyl-1-β-D-thiogalactopyranoside (IPTG) at 25 °C for 8 h. The recombinant construct pGEX4T-TEV-GST-CaMKK2 was expressed in the BL21 strain of *E. coli*, grown overnight, and expression was induced with 100 μM isopropyl-1-β-D-thiogalactopyranoside (IPTG) at 37 °C for 4 h. Bacteria were then harvested by centrifugation at 1000*g* for 20 min, and the cell pellet was resuspended in buffer (1X phosphate-buffered saline (PBS), pH 7.4, containing 2 mM ethylene diamine tetraacetic acid (EDTA), 10 mM dithiothreitol (DTT), 1% Triton X-100, and 1 mM phenylmethylsulfonyl fluoride (PMSF)). The cell suspension was sonicated, and the lysate was clarified by centrifugation at 16,000*g* for 30 min at 4 °C. The resulting supernatant was loaded onto glutathione-Sepharose beads, and the recombinant protein was isolated. The purity of all fusion proteins was evaluated by Coomassie staining of SDS-PAGE.

### Cell culture and transfection

HEK-293, HeLa, and HepG2 cells were obtained from the American Type Culture Collection. MEF cells derived from IQGAP1-null and normal littermate control mice have been previously described (19). HEK-293, HeLa, and MEF cell lines were cultured in Dulbecco's modified Eagle's medium (DMEM, Gibco) supplemented with 10% (v/v) fetal bovine serum. HepG2 cells were cultured in modified Eagle's minimum essential medium (EMEM, Gibco) supplemented with 10% (v/v) fetal bovine serum. HEK-293 cells were transfected with pEGFP-N1-CaMKK2, pEGFP-C1-AMPKα1 plasmids, or empty pEGFP vector using Lipofectamine 2000 transfection reagent according to the manufacturer’s instructions. MEF cells were transfected with pcDNA3-myc-IQGAP1 or pcDNA3 empty vector using Lipofectamine LTX transfection reagent according to the manufacturer’s instructions. HeLa or HepG2 cells were transfected with 5 μl of 10 μM IQGAP1 or control siRNA using Lipofectamine RNAiMAX following the manufacturer’s instructions. Where indicated, cells were treated for 15 min with 10 μM A23187 or DMSO as a vehicle control 72 h after siRNA transfection. Cells were harvested in lysis buffer, sonicated, and resolved by Western blotting. Membranes were probed with anti-IQGAP1, anti-pAMPK, anti-AMPK, anti-pCaMK1, antitubulin, and/or anti-CaMKK2 antibodies and visualized as described above. The amounts of pAMPK or pCaMK1 were quantified and corrected for the amounts of tubulin in the same samples.

For the combined knockdown and rescue experiments, HeLa cells were transfected with 5 μl of 10 μM siIQGAP1-8 or scrambled siRNA (control) and 10 μl Lipofectamine RNAi MAX in 200 μl Optimem per well in a 6-well plate. Cells were trypsinized, and 100,000 cells per well were added to 6-well dishes. After 24 h, HeLa cells were transfected with 2 μg of siRNA-resistant IQGAP1 plasmids or vector control using 6 μl Lipofectamine 2000 in 200 μl Optimem according to the manufacturer’s instructions. 72 h after siRNA transfection, cells were treated with DMSO or 10 μM A23187 for 15 min.

### T_N_T product production and binding analysis

Full-length IQGAP1, the N-terminal half (amino acids 2–864), the C-terminal half (865–1657), and the IQ region (717–916) were synthesized using the T_N_T quick coupled transcription and translation (T_N_T) system (Promega) essentially as described previously (62). Briefly, 1 μg of pcDNA3-IQGAP1, IQGAP1-N, IQGAP1-C, or IQGAP1-IQ was incubated with 40 μl of T_N_T Quick Master Mix (Promega) and 20 μCi of [^35^S]methionine (PerkinElmer) at 30 °C for 90 min. T_N_T products were diluted in lysis buffer and used in pull-down assays. Equal amounts of the T_N_T products were incubated with GST-AMPKα1, GST-CaMKK2, or GST alone for 3 h at 4 °C. Complexes were washed five times with lysis buffer and separated by SDS-PAGE. The gels were dried, and autoradiography was performed. Where indicated, T_N_T studies were performed using 1 μg of pcDNA3-IQGAP1 full-length or IQGAP1Δ746-860 incubated with 40 μl of T_N_T Quick Master Mix (Promega) with biotinylated-lysine at 30 °C for 90 min. Equal amounts of the biotinylated-T_N_T products were incubated with GST-AMPKα1, GST-CaMKK2, or GST alone for 3 h at 4 °C. Complexes were washed and separated by SDS-PAGE. The gel was cut at the 150-kDa region. The lower portion of the gel was stained with Coomassie Blue. The top part was transferred to a PVDF membrane, blocked with blocking buffer for 1 h at 22 °C, and then incubated with IRDye-conjugated streptavidin for 1 h at 22 °C. After washing, complexes were detected using the Odyssey Imaging System (LI-COR).

### Immunoprecipitation

Immunoprecipitation was performed essentially as previously described (20). Briefly, cells were plated in 10-cm dishes to obtain 80% confluence. The following day, cells were transfected with GFP-CaMKK2, GFP-AMPKα1, or GFP alone. After 48 to 72 h, the cells were washed with ice-cold PBS and lysed in lysis buffer (50 mM Tris-HCl, pH 7.4, 150 mM NaCl and 1% (v/v) Triton X-100) with Halt protease inhibitor cocktail. Lysates were subjected to sonication for 5 s, and the insoluble fraction was pelleted by centrifugation at 16,000*g* at 4 °C. Supernatants were precleared with glutathione-Sepharose beads for 1 h at 4 °C. Clarified cell lysates were equalized for protein concentration using the Bio-Rad Protein Assay Dye Reagent protein assay, and equal amounts of protein lysate were incubated with anti-IQGAP1 polyclonal antibodies for 3 h at 4 °C. Similar samples were incubated with NIRS as the negative control and were processed in parallel. Alternatively, GFP-tagged proteins were immunoprecipitated using GFP-Trap Agarose for 3 h following the manufacturer’s instructions. Immune complexes were isolated using protein A-Sepharose beads or using GFP-Trap beads, washed five times in lysis buffer, and resolved by Western blotting. Membranes were probed with anti-GFP and anti-IQGAP1 antibodies and were visualized as described above.

### Measurement of AMPK phosphorylation

Control and IQGAP1-null MEFs were plated at 10^5^ cells per well in a 6-well dish and allowed to attach overnight. The following day, cells were incubated with 5 mM metformin for 4 h or 10 μM A23187 for 15 min in DMEM with 10% FBS at 37 °C. DMSO was used as a control for A23187. Cells were harvested in lysis buffer, sonicated, and resolved by Western blotting as described above. Membranes were probed with anti-IQGAP1, anti-pAMPK, anti-AMPK, antitubulin, and/or anti-CaMKK2 antibodies and visualized as described above. The amounts of pAMPK were quantified and corrected for the amounts of tubulin in the same samples.

### Animals

IQGAP1-null (36) and wild-type (littermate control) mice were bred in the animal facility at the National Institutes of Health (NIH) and maintained according to NIH guidelines. The studies were performed with approval of the NIH Animal Care and Use Committee. Wild-type or IQGAP1-null male mice (14–18 weeks old) were fasted for 16 h with water available *ad libitum*, then mice were euthanized with carbon dioxide. The liver and epididymal fat pads were harvested and frozen immediately.

### RNA extraction

100 mg of tissue was placed in a homogenizing tube containing CKMix beads (catalogue number 10409, Bertin) and 1 ml of TRIzol Reagent. Tissues were subjected to centrifugation at 6500 rpm for 20 s using a Precellys homogenizer (Bertin). Samples were subjected to centrifugation for 5 min at 12,000*g* at 4 °C. 200 μl of chloroform was added to the supernatant. After incubation for 3 min at 22 °C, the samples were subjected to centrifugation for 15 min at 12,000*g* at 4 °C. The aqueous phase containing the RNA was transferred to a new tube, and 500 μl of chloroform was added. After incubation for 10 min at 22 °C, samples were subjected to centrifugation for 10 min at 12,000*g* at 4 °C. The pellets were resuspended in 1 ml of 75% ethanol and transferred to an RNeasy Mini spin column (catalogue number 74104, QIAGEN). RNA was extracted following the manufacturer’s instructions.

### Quantitative RT-PCR

Total RNA isolated from mouse tissues was used for quantitative RT-PCR. 1 μg of RNA was reverse transcribed to cDNA using a High Capacity cDNA Reverse Transcriptase kit (catalogue number 4368814, Applied Biosystems) according to the manufacturer’s instructions. RT-PCR was performed on a StepOnePlus Real Time PCR system (Applied Biosystems) using SYBR Green PCR Master Mix (catalogue number 4309155, Applied Biosystems) and 200 nM forward and reverse primers, essentially as previously described (63). The primers used are described in Table 2. RT-PCR enzyme activation was initiated for 10 min at 95 °C and then amplified for 40 cycles (15 s at 95 °C and 1 min at 60 °C). The fold change in mRNA expression was calculated by the delta–delta CT method with StepOnePlus software (Applied Biosystems). Expression of genes was normalized to the housekeeping gene 18S. Each assay was performed in triplicate.

**Table 2:** Primers used in this study

Gene detected	Forward primer	Reverse primer	Reference
PCK1	CTGCATAACGGTCTGGACTTC	CAGCAACTGCCCGTACTCC	(64)
G6PC	TTCAAGTGGATTCTGTTTGG	AGATAGCAAGAGTAGAAGTGAC	(65)
ACC1	ATGGGCGGAATGGTCTCTTTC	TGGGGACCTTGTCTTCATCAT	(64)
FASN	GGAGGTGGTGATAGCCGGTAT	TGGGTAATCCATAGAGCCCAG	(64)
18S RNA	GCAATTATTCCCCATGAACG	GGCCTCACTAAACCATCCAA	(66)

### Statistical analysis

Western blots were quantified with Image Studio 2.0 (LI-COR) according to the manufacturer’s instructions. After quantifying the bands, we calculated the ratio of each pAMPK band to the relevant loading control band (tubulin) in the same sample. We normalized data by dividing each ratio by the ratio of control (untreated, vector-transfected, or endogenous IQGAP1 expressing cells) to its loading control. Thus, control cells equaled 1. Statistical analysis of AMPK signaling was performed by repeated measures one-way ANOVA with a Geisser–Greenhouse correction using Prism7 (GraphPad). All analyses were corrected with the Tukey post-hoc test. Statistical analysis of mRNA expression was performed by unpaired *t*-test with Welch’s correction using Prism7. Wild-type fed mice were set as 1.

## Data availability

All data are contained in the article.

## Conflict of interest

The authors declare that they have no conflicts of interest with the contents of this article.
